# Genome and Transcriptome Analyses Facilitate Genetic Control of *Wohlfahrtia magnifica*, a Myiasis-Causing Flesh Fly

**DOI:** 10.3390/insects14070620

**Published:** 2023-07-10

**Authors:** Zhipeng Jia, Surong Hasi, Deng Zhan, Bin Hou, Claus Vogl, Pamela A. Burger

**Affiliations:** 1 Research Institute of Wildlife Ecology, Department of Interdisciplinary Life Sciences, University of Veterinary Medicine Vienna, Savoyenstrasse 1, 1160 Vienna, Austria; 2 Key Laboratory of Clinical Diagnosis and Treatment Technology in Animal Disease, Ministry of Agriculture and Rural Affairs, Inner Mongolia Agricultural University, Hohhot 010018, China; 3 Institute of Animal Breeding and Genetics, Department of Biomedical Sciences, University of Veterinary Medicine Vienna, Veterinaerplatz 1, 1210 Vienna, Austria

**Keywords:** *Wohlfahrtia magnifica*, myiasis, *tra* gene, *tra2* gene, Iso-Seq, RNA-seq, genetic control

## Abstract

**Simple Summary:**

*Wohlfahrtia magnifica*, a flesh fly, parasitizes several warm-blooded vertebrates and causes severe traumatic myiasis, detrimental to animal welfare and the livestock industry across Eastern and Southern Europe, Northern Africa, and Western and Northeast Asia. Genetic control has emerged as an effective and promising alternative to insecticides for controlling insect pests. In this study, we isolated and characterized two sex-determination genes, *W. magnifica transformer (Wmtra)* and *W. magnifica transformer2* (*Wmtra2*). These investigations may contribute to the establishment of genetically modified strains in *W. magnifica*. For example, the regulated first intron of *Wmtra*, a key component in the conditional female lethal transgenic systems, can be used to control the sex-specific expression of a pro-apoptotic gene, as developed for myiasis-causing blow flies, *Lucilia cuprina* and *Cochliomyia hominivorax*. Additionally, we performed a differential expression gene analysis between adult males and adult females and identified five candidate genes (*vasa* (*vas*), *nanos* (*nanos*), *bicoid* (*bcd*), *Bicaudal C* (*BicC*), and *innexin5* (*inx5*)) from the female-biased gene set that could upregulate Cas9 expression in the germline in Cas9-based homing gene drive systems, as established in mosquitoes. In summary, the isolation and characterization of these genes provide a solid foundation for the development of genetic control programs against *W. magnifica*.

**Abstract:**

Myiasis caused by *Wohlfahrtia magnifica* is a widespread parasitic infestation in mammals. The infested host suffers from damage as the developing larvae feed on its tissues. For the control of myiasis infestation, genetic methods have been shown to be effective and promising as an alternative to insecticides. Combining genome, isoform sequencing (Iso-Seq), and RNA sequencing (RNA-seq) data, we isolated and characterized two sex-determination genes, *W. magnifica transformer (Wmtra)* and *W. magnifica transformer2* (*Wmtra2*), whose orthologs in a number of insect pests have been utilized to develop genetic control approaches. *Wmtra* transcripts are sex-specifically spliced; only the female transcript encodes a full-length functional protein, while the male transcript encodes a truncated and non-functional polypeptide due to the presence of the male-specific exon containing multiple in-frame stop codons. The existence of five predicted TRA/TRA2 binding sites in the male-specific exon and the surrounding intron of *Wmtra*, as well as the presence of an RNA-recognition motif in WmTRA2 may suggest the auto-regulation of *Wmtra* by its own protein interacting with WmTRA2. This results in the skipping of the male-specific exon and translation of the full-length functional protein only in females. Our comparative study in dipteran species showed that both the WmTRA and WmTRA2 proteins exhibit a high degree of similarity to their orthologs in the myiasis-causing blow flies. Additionally, transcriptome profiling performed between adult females and adult males reported 657 upregulated and 365 downregulated genes. Functional analysis showed that among upregulated genes those related to meiosis and mitosis Gene Ontology (GO) terms were enriched, while, among downregulated genes, those related to muscle cell development and aerobic metabolic processes were enriched. Among the female-biased gene set, we detected five candidate genes, *vasa* (*vas*), *nanos* (*nanos*), *bicoid* (*bcd*), *Bicaudal C* (*BicC*), and *innexin5* (*inx5*). The promoters of these genes may be able to upregulate Cas9 expression in the germline in Cas9-based homing gene drive systems as established in some flies and mosquitoes. The isolation and characterization of these genes is an important step toward the development of genetic control programs against *W. magnifica* infestation.

## 1. Introduction

*Wohlfahrtia magnifica* (Schiner, 1862; Diptera, Sarcophagidae) is an obligate parasitic species belonging to the group of flesh flies that cause severe myiasis in livestock, such as horses [1,2], sheep [3,4], camels [5,6], and even in humans [7,8]. Similar to other myiasis-causing flies, female adults of *W. magnifica* are attracted by wounds or natural body orifices of the host, such as the genitalia, and deposit the first instar larvae there. For subsequent development, the first- to third-stage larvae feed on the tissues, leading to serious health consequences for the host.

In regions where *W. magnifica* is distributed, from Eastern and Southern Europe and Northern Africa to Western and Northeast Asia [6,9,10,11,12,13,14,15,16,17], *W. magnifica*-related myiasis has led to important animal welfare and health problems, as well as huge economic losses due to reproduction problems, lameness, blindness, and even death if the infestation stays untreated [9,18,19]. As of now, a large number of cases of myiasis resulting from *W. magnifica* have been documented. For example, in Spain, Remesar et al. investigated a total of 73,683 sheep from 122 flocks in Albacete Province, and the results indicated the overall flock prevalence of traumatic myiasis was 95.9%, with an individual prevalence of 7.1% [20]; in China, Liu et al. surveyed 2038 female camels in selected sites from May to October 2021 in Inner Mongolia, and the results showed that the overall prevalence rate was 26.6% [21]. Killing the larvae with insecticides is the method most employed to fight myiasis-causing flies infestation. But frequent use of insecticides can result in resistance, necessitating an increase in the insecticide dosage until it eventually loses its efficacy. Furthermore, it is toxic to beneficial insects and non-target species in the local environment. Long-term prevention of *W. magnifica* and other myiasis-causing fly infestation is not reliably achieved using insecticides. For example, *Lucilia cuprina*, a myiasis-causing blow fly in Australia and New Zealand, has developed resistance to a wide range of insecticides by metabolic and target site insensitivity-resistance mechanisms [22].

Genetic control holds significant potential in effectively and promisingly managing insect pests. This approach aims to suppress the population size of target pest species to a non-critical level through targeting their reproductive capacity. Developed in the 1950s by Raymond Bushland, Edward Knipling, and colleagues [23,24], the sterile insect technique (SIT) is the best-known, as well as very successful, genetic control strategy. For example, *Cochliomyia hominivorax*, a blow fly that is an obligatory myiasis agent, has been successfully eradicated in North and Central America using SIT [25,26]. According to its guiding principles, SIT entails mass-rearing insects in special facilities, subjecting them to a high dosage of ionizing radiation, and dispersing them widely in predetermined regions. As a result, sterile male flies can mate with females from a wild population, resulting in no offspring being produced and further declines in insect populations over several generations. Traditionally, both sexes are released. However, the co-released females can compete with the wild females for mating with the released sterile males, which can increase the number of insects required for population suppression. In field tests with sterilized insect pests, such as the Mediterranean fruit fly in Guatemala, releasing male-only SIT may be three to five times more effective than the bisexual release in reducing the targeted populations [27].

Conditional female lethal transgenic strains for the myiasis-causing flies *C. hominivorax* [28] and *L. cuprina* [29] were produced considering the advantages of male-only releases and were found to be highly effective. The system consists of a driver construct expressing the *tetracycline transactivator* (*tTA*) gene under the control of a promoter, and an effector construct composed of a tTA-regulated pro-apoptotic gene, such as the *head involution defective* (*hid)* gene. When adding the antidote tetracycline to the diet, tetracycline can bind to tTA and thereby prevent the expression of the pro-apoptotic gene. In contrast, when tetracycline is absent, tTA can bind to a tetracycline operator (tetO), promoting the expression of the pro-apoptotic gene. To make the system sex-specific, the sex-specifically spliced first intron of *tra* is introduced within the pro-apoptotic gene. As a result, only females die when insects are reared with a diet lacking in tetracycline, while both females and males can survive with a diet containing tetracycline.

As another potential approach for insect pest control, Cas9-based homing gene drives have been established in a variety of pest species, in particular the mosquitoes *Anopheles gambiae*, *Anopheles stephensi*, and *Aedes aegypti* [30,31,32,33,34]. In its simplest form, the “homing construct” system consisting of a Cas9 nuclease and a guide RNA (gRNA) is designed to insert precisely into the genome. The Cas9 nuclease is guided by a gRNA to cleave a target site on the wild-type chromosome and form the double-strand break. Subsequently, taking the locus incorporating the homing construct as a template, the double-strand break can undergo homology-directed repair (HDR), a naturally occurring nucleic acid repair process. By copying similar sequences, this repair mechanism can result in the perfect copying of the drive allele containing the homing construct into the wild-type chromosome and effectively converting a heterozygote into a homozygote. Referred to as “super-Mendelian” inheritance, the frequency of transmitting the drive allele to the next generation is greater than expected by random segregation of heterozygous alleles, potentially enabling it to suppress pest populations. In a Cas9-based homing gene drive system, the selection of gene drive targets is essential. As the reproductive capacity of female flies determines the growth of insect populations, female development or reproduction genes could be outstanding candidates. For example, Carrami et al. generated a Cas9-based homing gene drive strain targeting the *tra* gene and showed its high efficiency for sex conversion from females to males in *D. melanogaster* [35].

In the sex determination pathway of some dipteran species, the TRA–TRA2 complex autoregulated the female-specific splicing of *tra* pre-mRNA and directed the splicing of the pre-mRNA of the transcription factor *doublesex* (*dsx*), whose protein, DSX, promotes sexual development by regulating the transcription of sex-specific differentiation genes. In addition, *tra* in the myiasis-causing blow flies, *L. cuprina* and *C. hominivorax*, has been used to create a conditional female lethal transgenic strain as it is responsible for turning sex-specific expression of a pro-apoptotic gene on or off. *Tra2* is often used as a target gene for genetic control strategies of insect pests. In this study, based on genome, isoform sequencing (Iso-Seq), and RNA sequencing (RNA-seq) data, we isolated and characterized two sex-determination genes, *W. magnifica transformer (Wmtra)* and *W. magnifica transformer2* (*Wmtra2*), with the aim of laying the foundation for the development of a conditional female lethal transgenic strain. From the female-biased gene set, we identified five candidate genes, *vasa* (*vas*), *nanos* (*nanos*), *bicoid* (*bcd*), *Bicaudal C* (*BicC*), and *innexin5* (*inx5*), whose promoters can drive Cas9 expression in the germline in Cas9-based homing gene drive systems, as established in some flies and mosquitoes. The isolation of these genes is an important step toward the development of genetic control programs for *W. magnifica* infestation.

## 2. Materials and Methods

### 2.1. Genome Resources of W. magnifica

In a previous publication [36], we reported sequencing, assembling, and annotating the genome of *W. magnifica*. The genome was deposited in GeneBank with accession number JAKWBJ000000000 under BioProject PRJNA778059. In addition, the annotation file and the putative transcripts and proteins of the genome of *W. magnifica* are available on Dryad (https://doi.org/10.5061/dryad.qfttdz0j8, accessed on 5 May 2022).

### 2.2. W. magnifica Sample Collection

In the study, the research species, *W. magnifica*, is an invertebrate agricultural insect pest, which is not an endangered or protected species. Second-stage and third-stage larvae samples of *W. magnifica* were collected non-invasively from domestic Bactrian camels in the field in Siziwang Banner, Ulanqab City, Inner Mongolia, China, therefore no animal experimental or ethical permits were necessary. The experimental protocols for the flies followed the procedures of Inner Mongolia Agricultural University. In short, the third-stage larvae were divided into two parts, one of which was placed in a foam box containing local soil; the rest along with the second-stage larvae were dropped directly into liquid nitrogen and then stored in a refrigerator at −80 °C. Subsequently, a portion of the three-day-old pupae was picked out of the soil in the foam box, and stored in a refrigerator at −80 °C. After 14 days, when the remaining pupae emerged into adult flies, the sex was distinguished, and adult females and adult males were placed into the refrigerator at −80 °C.

### 2.3. RNA Isolation and Assessment

The total RNA of each sample was extracted with the RNA Easy Fast Tissue/Cell kit (Tiangen Biotech, Beijing, China) following the manufacturer’s instructions. The concentration, purity, and integrity of the extracted RNA were measured using NanoDrop (Thermo Fisher Scientific, Wilmington, DE, USA), Agilent 5400 (Agilent Technologies, Palo Alto, CA, USA), and 1% agarose gels. Qualified RNA samples were used for PacBio and Illumina library construction.

### 2.4. Illumina RNA-Seq Library Construction, Sequencing and Data Filtering

High-quality total RNA extracted from six samples, including three females and three males (each sample with one individual), was used for RNA-seq library preparation using the NEBNext^®^ Ultra RNA Library Prep Kit for Illumina (New England Biolabs, Ipswich, MA, USA) according to the manufacturer’s instructions. In brief, the polyA fraction (mRNA) was purified from total RNA using oligonucleotides (dT) magnetic beads. The purified mRNA was fragmented and cDNA synthesized followed by end repair, A-tailing, adapter ligation, and PCR amplification steps. The prepared library was evaluated using Agilent 2100 Bioanalyzer (Agilent Technologies, Palo Alto, CA, USA) and qualified libraries were sequenced on an Illumina NovaSeq platform. Clean reads were generated by removing the adaptor sequences, low-quality reads, contamination from Bactrian camel and rRNA and by keeping reads with a minimum length of 75 base pairs (bp) using BBduk in the BBTools toolset [37]. 

### 2.5. PacBio Iso-Seq Library Construction, Sequencing, and Data Processing 

For Iso-Seq, the total RNA of different developmental stages and sexes was pooled in equal amounts. Subsequently, mRNA was isolated and reverse-transcribed into full-length cDNA using the SMARTer PCR cDNA Synthesis Kit (Clontech, Palo Alto, CA, USA). Two SMRTbell libraries were constructed using the SMRTbell Express Template Prep Kit 2.0 (Pacific Biosciences, Menlo Park, CA, USA). The prepared libraries were sequenced on the PacBio Sequel II platform. 

Iso-Seq raw data stored in the BAM files were processed using the CCS program v6.4.0 (https://github.com/PacificBiosciences/ccs, accessed on 27 October 2022) with default parameters, and circular consensus sequences (CCS) were called. CCS reads containing the 5′ primer, the 3′ primer and, the polyA tail were processed by primer removal for generating the full-length (FL) reads using the lima program v2.6.0 (https://github.com/pacificbiosciences/barcoding/, accessed on 5 November 2022) with the parameters: --isoseq --dump-clips --peek-guess. Next, the refine module of the IsoSeq3 program v3.8.1 (https://github.com/PacificBiosciences/IsoSeq, accessed on 5 November 2022) was employed to identify and remove polyA tails and concatemers to generate full-length non-concatemer (FLNC) reads. FLNC reads were clustered to generate transcripts using the cluster module of the IsoSeq3 program v3.8.1. As a result, high-quality and low-quality isoforms were obtained.

The pbmm2 program v1.9.0 (https://github.com/PacificBiosciences/pbmm2, accessed on 5 November 2022), a minimap2 SMRT wrapper for PacBio Iso-Seq data, was applied to map high-quality isoforms onto the reference genome of *W. magnifica* [36]. With the mapping results, the redundant isoforms were collapsed using the collapse module of the IsoSeq3 program v3.8.1.

### 2.6. Isolation of the Wmtra and Wmtra2 Genes

We isolated the *Wmtra* and *Wmtra2* genes from the collapsed Iso-Seq transcript dataset. However, we did not obtain a full-length male transcript of the *Wmtra* gene, probably because the male-specific transcript is lower-expressed. Therefore, the male-specific transcript was reconstructed by aligning the three male RNA-seq data to the *W. magnifica* genome [36] using the HISAT2 program v2.2.1 [38] and feeding the output to the StringTie program v2.2.1 [39] for a genome-based assembly. The obtained *Wmtra* and *Wmtra2* transcripts were aligned to the *W. magnifica* genome [36] using the Minimap2 program v.2.24 [40] for genomic organization analysis.

### 2.7. Reverse Transcription Polymerase Chain Reaction (RT-PCR) Validation

The same batch of total RNA of both adult females and adult males with RNA-seq was used for RT-PCR. Based on the sequence obtained by Iso-Seq, we designed primers in 5′ untranslated region and the third common exon to amplify the sex-specific region using Primer3Plus (https://www.primer3plus.com/, accessed on 4 May 2023) and oligos were listed as follows: 

Wmtra-F: 5′-CGGGAAGGTTAGGCTGTAGC-3′;

Wmtra-R: 5′-CGCAGATGAGGGTGGAGAAG-3′.

RT-PCR analysis for *Wmtra* was performed using the PrimeScript™ One Step RT-PCR Kit Ver.2 (Takara, Dalian, China), in which RNA→cDNA→PCR reactions were amplified in a single reaction system. Following the protocol’s instruction, PrimeScript 1 Step Enzyme Mix, 2X 1 Step Buffer, WmtraF, WmtraR, total RNA, and RNase Free dH2O were added to a 50 μL reaction system to amplify the sex-specific region under the condition of 1 cycle of 50 °C for 30 min and 94 °C for 2 min; 30 cycles of 94 °C for 30 s, 60 °C for 30 s, and 72 °C for 1 min. RT-PCR products were visualized on gel electrophoresis and then sent for Sanger sequencing. 

Since we detected two sequences of *Wmtra2* in the collapsed Iso-Seq dataset, we designed three pairs of primers to investigate whether both sequences are verifiably transcribed in *W. magnifica* and whether *Wmtra2* is sex-specific. The primer pair 1 include the start and the stop codons or regions in their close proximity; the primer pair 2 was designed by moving outwards. As both sequences were identical, except that it was 129 bp longer at the 3′ terminal ends, we designed the reverse primer Wmtra2-R3 of the primer pair 3 within this fragment to verify whether the longer sequence was present in *W. magnifica*. The primers for the *Wmtra2* amplification were as follows:

Wmtra2-F1: 5′-ATGAGTCCTCGTTCACGCAG-3′;

Wmtra2-R1: 5′-ACTGACACACTTCAAGGGGC-3′;

Wmtra2-F2: 5′-ACGGCTTTGCTTTTGTACAGT-3′;

Wmtra2-R2: 5′-ATGCATATGGTTCGATGGAATAAAT-3′;

Wmtra2-F3: 5′-TGGCGAAATTGAACATTTACGGA-3′;

Wmtra2-R3: 5′-AATTTCTTTCAAGTCTTTATTTTGCCT-3′.

Since we did not obtain the expected *wmtra2* product using the one-step RT-PCR method, we amplified *wmtra2* using a two-step approach, where reverse transcription and PCR are reacted in separate tubes. PCR reaction conditions were set to 1 cycle of 98 °C for 2 min; 35 cycles of 98 °C for 20 s, 55 °C for 20 s and, 72 °C for 30 s; 1 cycle of 72 °C for 5 min; and 1 cycle of 16 °C for 2 min.

### 2.8. Sequence Analysis

A multiple alignment of protein sequences was performed using Clustal Omega [41]; the analysis of the alignment results was performed with Jalview v.2.11.2.6 [42]. Phylogenetic analysis was carried out using the neighbor-joining method in the MEGA program v.11.0.13 [43] with 1000 bootstrap replicates. Accession numbers for TRA sequence analysis used in this study are *Lucilia sericata* (AGE31795.1), *L. cuprina* (ACS34687), *C. hominivorax* (AGE31793.1), *Cochliomyia macellaria* (AGE31794.1), *Bactrocera oleae* (CAG29241.1), *Ceratitis capitata* (XP_004526947.1), *Musca domestica* (ACY40709.1), *Drosophila melanogaster* (AAF49441.1), and *Drosophila virilis* (EDW68645.2). Accession numbers for TRA2 sequence analysis used in this study include *L. cuprina* (ACS34688.1), *C. hominivorax* [44], *L. sericata* (XP_037815979.1), *C. capitata* (ACC68674.1), *M. domestica* (AAW34233.1), *B. oleae* (CAD67988.1), *D. melanogaster* (AAA28953.1), *Drosophila suzu*kii (ATI14861.1), *D. virilis* (EDW60892.2), *Stomoxys calcitrans* (NP_001298164.1), *Bactrocera correcta* (AJE26246.1), *Anastrepha bistrigata* (CBJ17289.1), and *Anastrepha obliqua* (CBJ17280.1).

### 2.9. Identification of Differentially Expressed Genes (DEGs)

We used three adult female and three adult male samples to investigate DEGs. The clean reads of each sample were mapped to the genome of *W. magnifica* [36] using the HISAT2 program v. 2.2.1 [38]. With the aligned bam files as input, raw counts of each sample were generated with the featureCounts program v2.0.3 [45]. In addition, raw count values were normalized the transcript per million (TPM). Prior to differential gene expression analysis, we also conducted a principal component analysis (PCA) after regularized log transformation (rlog) of TPM by the rlog function of the DESeq2 R package [46]. Subsequently, a differential expression analysis of genes was performed with the DESeq2 R package [46] using a *q*-value of < 0.05 and fold change ≥ 2 as a cutoff for the assignment of DEGs. GO enrichment analysis of DEGs was conducted with a cut-off criterion of *q*-value <  0.05.

### 2.10. Promoter Analysis

We extracted the upstream sequences of the start codon of *Wmnanos* by 2000 bases as a regulatory region harboring the promoter. The transcription start site and the putative TATA box were identified with BDGP (https://www.fruitfly.org/seq_tools/promoter.html, accessed on 19 May 2023). We used AliBaba2.1 (http://gene-regulation.com/pub/programs/alibaba2/, accessed on 19 May 2023) to predict transcription factor binding sites. AliBaba2.1 was set to the default settings except for Pairsim and Matrix conservation which were set to 64 and 80%, respectively.

### 2.11. Identification of Target Genes against W. magnifica Infestation

We followed the approach of Anstead et al. [47], who exploited functional genomic data of the extensively studied fruitfly *D. melanogaster* as a resource and inferred the functions of 988 genes of *L. cuprina*, whose orthologs in *D. melanogaster* were single-copy and associated with (semi-)lethality. In our study, we used the same 988 protein sequences in *D. melanogaster* as a query to search against the protein set of *W. magnifica* with the BLASTP program v2.7.1 (E-value ≤ 1 × 10^−20^). If an ortholog of these proteins was detected in *W. magnifica*, we considered it as a potential target for the development of vaccines, drugs, or genetic control measures.

## 3. Results

### 3.1. Isolation and Characterization of the Wmtra Gene

**Figure 1:**
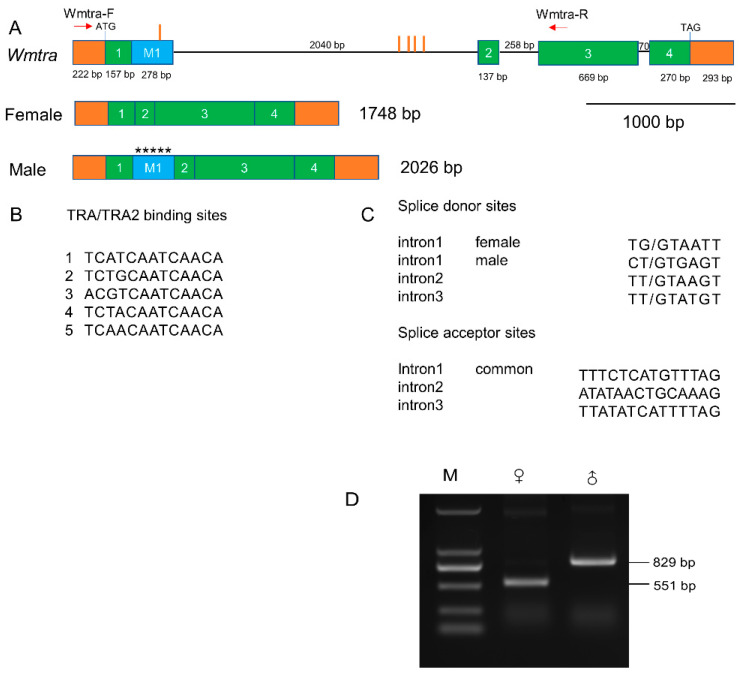
Genomic organization and sex-specific transcripts of *Wmtra*. (**A**) The *Wmtra* gene on the top diagram consists of four common exons 1, 2, 3, and 4 in both female and male transcripts (green boxes) and a male-specific exon M1 (blue box). Introns are represented by black horizontal lines. The 5′ and 3′ untranslated regions are shown in orange boxes. The translational start and stop locations are marked, and exon and intron lengths are shown in bp. The red vertical lines indicate the locations of the putative TRA/TRA2 binding sites in and around the exon M1. The red arrows indicate the primers. Transcripts for males and females are shown below the gene. Five asterisks in the exon M1 represent multiple in-frame stop codons (TAA, TGA, TAG, TAA, and TAG). (**B**) Sequences of the five TRA/TRA2 binding sites identified in the genomic DNA of the *Wmtra* gene. (**C**) Splice donor and acceptor sites of all *Wmtra* introns. The intron 1 “female” donor site is used to produce the female transcript and the intron 1 “male” donor site is used for males. The intron 1 “common” acceptor site is used to produce both female and male transcripts. (**D**) The detection of sex-specific transcripts of the *Wmtra* gene by RT-PCR analysis. M, ♀ and ♂ indicate the marker, adult females and adult males, respectively.

Based on the Iso-Seq data, we successfully identified a female full-length transcript of the *Wmtra* gene of 1748 bp (Figure 1A and Supplementary Materials File S1). It consists of an open reading frame encoding 410 amino acids (Supplementary Materials File S1), as well as a 222 bp long 5′ untranslated region and a 293 bp long 3′ untranslated region. We also reconstructed (see Section 2.6) a male transcript of 2026 bp from the genome-based assembly of the male RNA-seq data (Figure 1A and Supplementary Materials File S1). The male transcript encodes a short protein of 63 amino acids (Supplementary Materials File S1), which is truncated and non-functional, because of the absence of the serine-arginine dipeptide-rich region (RS domain) involved in protein–protein interactions. Transcript differences of *Wmtra* between males and females result from a similar sex-specific splicing pattern (Figure 1A) as in the blow flies *C. hominivorax* [48] and *L. cuprina* [49]. 

The PCR verification results showed a 551 bp RT-PCR product in female flies, while in males we detected an 829 bp product, the extra 278 bp being the male-specific exon, which is consistent with the sequencing results (Figure 1D).

The *Wmtra* gene includes five exons and three introns (Figure 1A). The exons 1–4 are common in the transcripts of females and males, while the exon M1 is male-specific, containing multiple in-frame translation stop codons (Figure 1A). Except for different splice donor sites in the first intron, the splicing pattern between the male and female transcript is identical (Figure 1A,C). The exon M1 is located between the common exons 1 and 2 and is contiguous with the common exon 1 (Figure 1A).

Within the *Wmtra* sequence, five TRA/TRA2 binding sites were identified (Figure 1A,B). Among them, four clustered sites are located in the first intron and one in the exon M1. 

**Figure 2:**
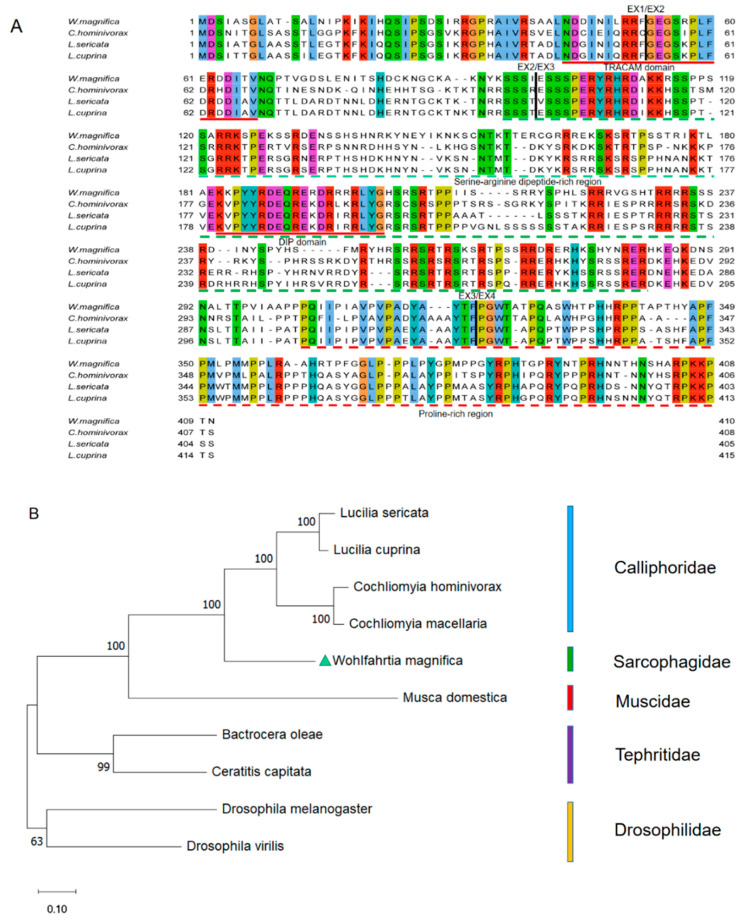
Multiple sequence alignments and phylogenetic analysis of TRA proteins among *W. magnifica* and other dipteran species. (**A**) Multiple sequence alignment of TRA proteins from *W. magnifica*, *C. hominivorax*, *L. sericata* and *L. cuprina*. Identical amino acids are shaded in the same color. The corresponding positions of the exon/intron boundaries are indicated in the TRA proteins by black vertical lines. The red horizontal lines represent the TRACAM domain and the DIP domain. The green and red horizontal dotted lines represent the serine-arginine dipeptide-rich region and the proline-rich region, respectively. (**B**) The neighbor-joining tree of selected TRA proteins from dipteran species. The numbers represent bootstrap support values from 1000 replicates. The green triangle highlights *W. magnifica*.

The multiple alignments of protein sequences indicate that the first, second, and third introns occur at identical positions in *Wmtra*, *Lctra, Chtra*, and *Lstra* (Figure 2A). In addition, we found up to 50.26%, 50.95%, and 52.76% identities between the WmTRA and LcTRA, ChTRA, and LsTRA proteins, respectively. WmTRA contains a characteristic serine-arginine dipeptide-rich region (RS domain) and a proline-rich region at the C-terminal end (proline-rich domain) (Figure 2A). In addition, a TRACAM (C, *Ceratitis*; A, *Apis*; M, *Musca*) domain and a conserved DIP (DIPTERA) domain in dipteran species were identified (Figure 2A). The phylogenetic analysis shows that the WmTRA protein and Calliphoridae TRA proteins form a cluster and are more closely related to each other than to Muscidae, Tephritidae, or Drosophilidae species (Figure 2B).

### 3.2. Isolation and Characterization of the Wmtra2 Gene

**Figure 3:**
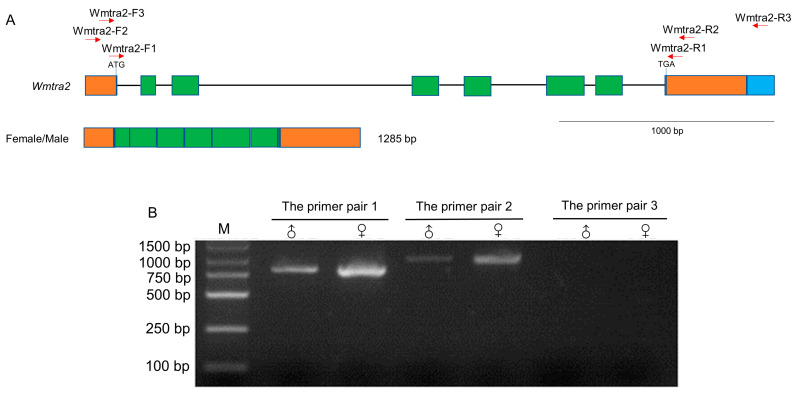
Genomic organization and the transcript of *Wmtra2*. (**A**) The *Wmtra2* gene on the top diagram consists of 8 exons. The green and orange regions represent coding regions and untranslated regions, respectively. Introns are represented by black horizontal lines. The blue region represents the difference between two sequences of *Wmtra2* from the Iso-Seq dataset. The translational start and stop locations are marked. The red arrows indicate the primers. The transcript for females/males is shown below the gene. (**B**) The detection of the transcript of the *Wmtra2* gene by RT-PCR analysis. M, ♀ and ♂ indicate the marker, adult females and adult males, respectively.

The *Wmtra2* gene contains eight exons and seven introns (Figure 3A). The putative start codon is located at the last three bases of the first exon and the stop codon is in the 15th–17th bases of the eighth exon. Two sequences of *Wmtra2* with 1285 bp and 1414 bp in length (Supplementary Materials File S2) were found from the collapsed Iso-Seq dataset. Except for the difference in length of the 3′ terminal end, the other regions of the two sequences are identical, encoding a putative protein of 258 amino acids (Supplementary Materials File S2).

The PCR validation results showed that the amplification products using primer pair 1 and primer pair 2 were 798 and 953 bp in length, respectively, and the product lengths are consistent in male flies and female flies (Figure 3B). In contrast, using the primer pair 3, there was no PCR product (Figure 3B), suggesting that *Wmtra2* does not transcribe the 1414 bp long sequences, which may be a redundancy in the Iso-Seq dataset. This is consistent with *L. cuprina*, which transcribes a single non-sex-specific transcript.

**Figure 4:**
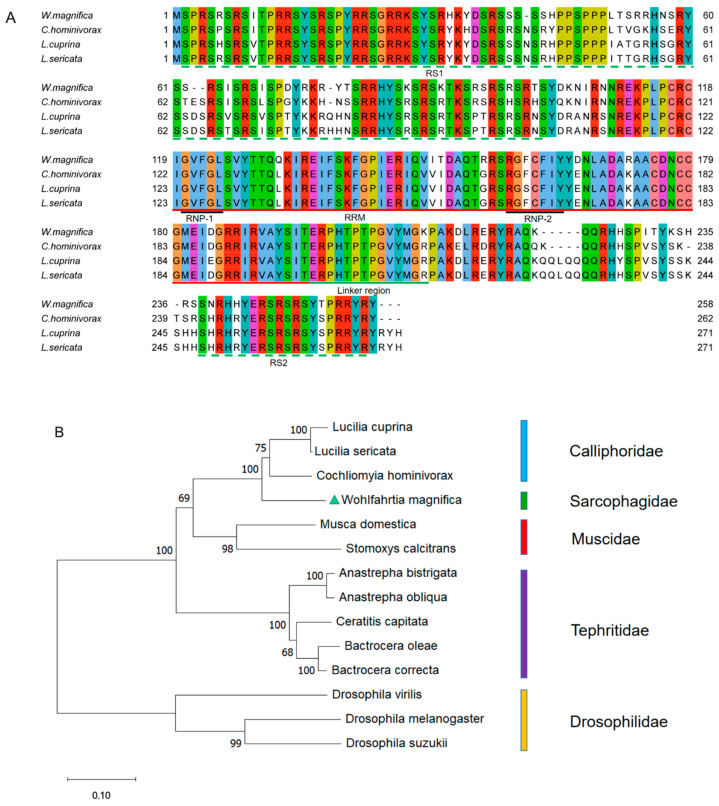
Multiple sequence alignments and phylogenetic analysis of TRA2 proteins among *W. magnifica* and other dipteran species. (**A**) Multiple sequence alignments of TRA2 proteins from *W. magnifica*, *C. hominivorax*, *L. cuprina*, and *L. sericata*. Identical amino acids are shaded in the same color. The red, black, and green horizontal lines represent RRM, RNP, and the linker region, respectively. The green horizontal dotted lines represent the RS1 domain and the RS2 domain. (**B**) The neighbor-joining tree of selected TRA2 proteins from dipteran species. The numbers represent bootstrap support values from 1000 replicates. The green triangle highlights *W. magnifica*.

The multiple alignment of protein sequences between the WmTRA2 protein and the TRA2 proteins from other myiasis-causing flies shows that the WmTRA2 protein contains an RNA-recognition motif (RRM) with two ribonucleoprotein regions (RNP1 and RNP2) immediately followed by the linker region and flanked by a serine-arginine dipeptide-rich N-terminal region (RS1 domain) and a serine-arginine dipeptide-rich C-terminal region (RS2 domain), which mediate protein–protein interactions (Figure 4A). The RS1 domain is mainly encoded by exons 2, 3, and 4, the RRM domain by exons 5 and 6, and the RS2 domain by exons 7 and 8. Phylogenetic analysis between the WmTRA2 protein and the TRA2 proteins from other dipteran species shows that, similarly to WmTRA, the WmTRA2 protein clusters with TRA2 proteins in Calliphoridae as these species belong to Oestroidea (Figure 4B).

### 3.3. Gene Expression Analysis between Adult Females and Adult Males

After removing low-quality reads, adaptors, rRNA, and contaminants, a total of 38.66 Gb clean paired-end reads, including 18.44 Gb from females and 20.22 Gb from males, were used for downstream analyses (Supplementary Materials Table S1). The number of clean reads per sample varies between 39,186,716 and 47,109,424 among the six sequenced samples (Supplementary Materials Table S1). The PCA results show that the biological replicates of male and female samples are distributed in two separate groups (Supplementary Materials Figure S1). In addition, more than 92% of clean reads from each sample can be mapped to the *W. magnifica* genome (Supplementary Materials Table S1).

**Figure 5:**
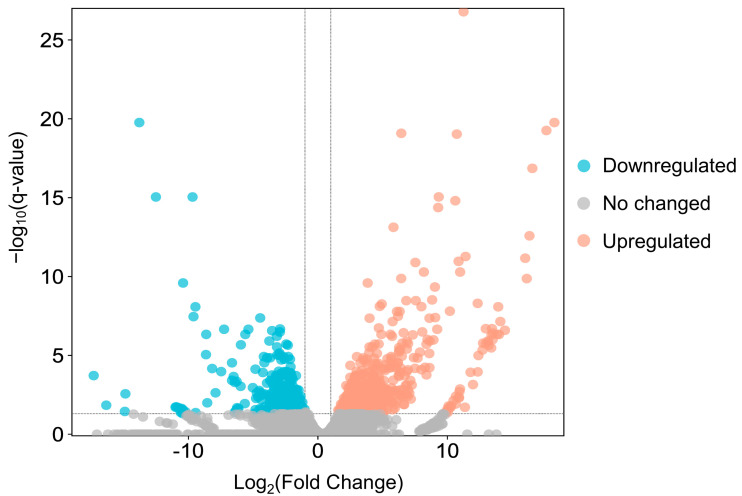
The volcano plot of DEGs in adult females versus adult males. Red dots and blue dots indicate upregulated genes and downregulated genes, respectively; grey dots are genes whose expression levels do not reach statistical significance.

**Figure 6:**
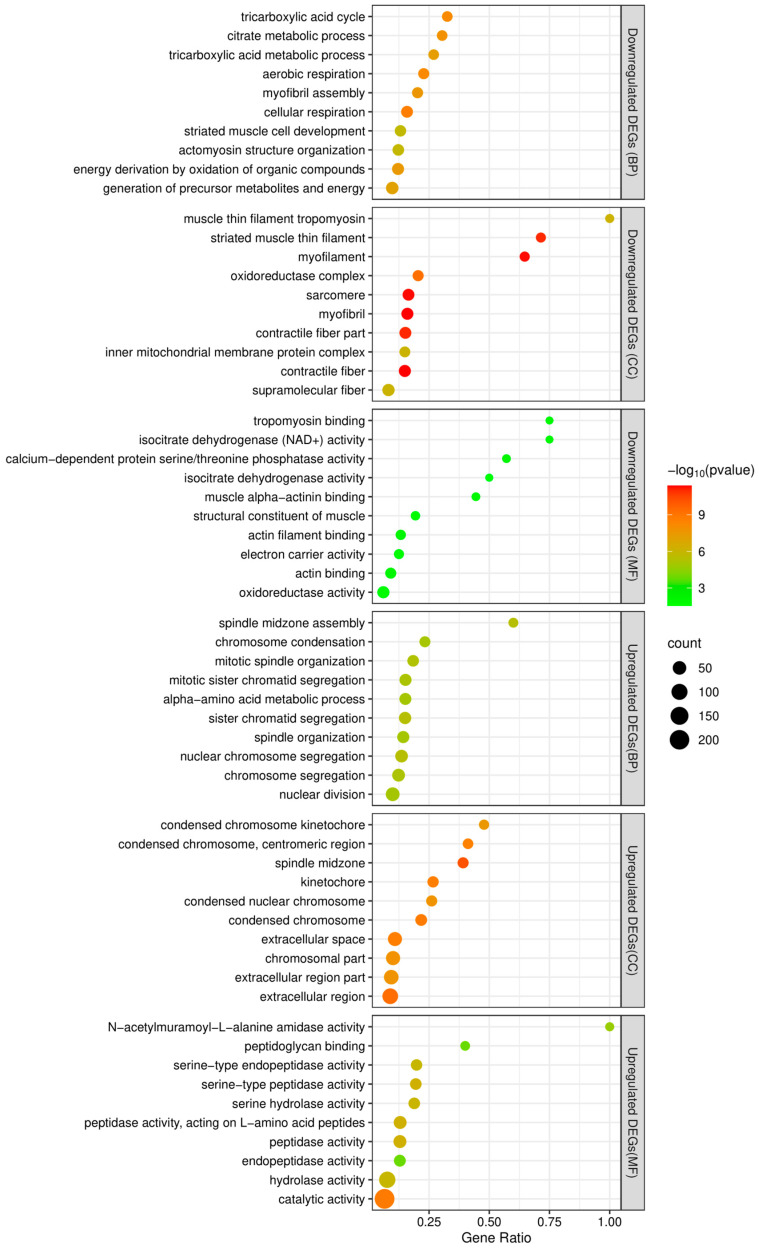
The top 10 enriched GO terms of functional enrichment analyses of downregulated and upregulated DEGs. The x-axis indicates the gene ratio and the y-axis represents the different GO terms. BP, CC, and MF represent Biological Process, Cellular Component, and Molecular Function groups of GO, respectively.

Between adult males and adult females, 1022 genes were found to be differentially expressed, of which 365 were downregulated and 657 upregulated in females (Figure 5 and Supplementary Materials Table S2). The top 10 terms of a subsequent GO enrichment analysis using DEGs are shown in Figure 6. We noted that genes upregulated in males are annotated for GO terms involved in muscle and mitochondrial structure (e.g., Cellular Component: “striated muscle thin filament” and “mitochondrial membrane”) and in muscle cell development and aerobic metabolic processes (e.g., Biological Process: “striated muscle cell development”, “muscle contraction”, “striated muscle cell differentiation”, and aerobic respiration) (Supplementary Materials Table S3). On the other hand, genes upregulated in females are annotated for GO terms involved in mitosis and meiosis (e.g., Cellular Component: “spindle midzone” and “condensed chromosome”; Biological Process: “chromosome segregation” and “nuclear division”) (Supplementary Materials Table S3). 

### 3.4. Candidate Genes for Cas9-Based Homing Gene Drive

From the female-biased gene set, several maternally expressed genes important for fertility (*inx5*) or embryonic development (*vas*, *nos*, *bcd*, and *BicC*) were identified, whose promoters could be used to drive Cas9 expression in the germline in Cas9-based homing gene drive systems.

**Figure 7:**
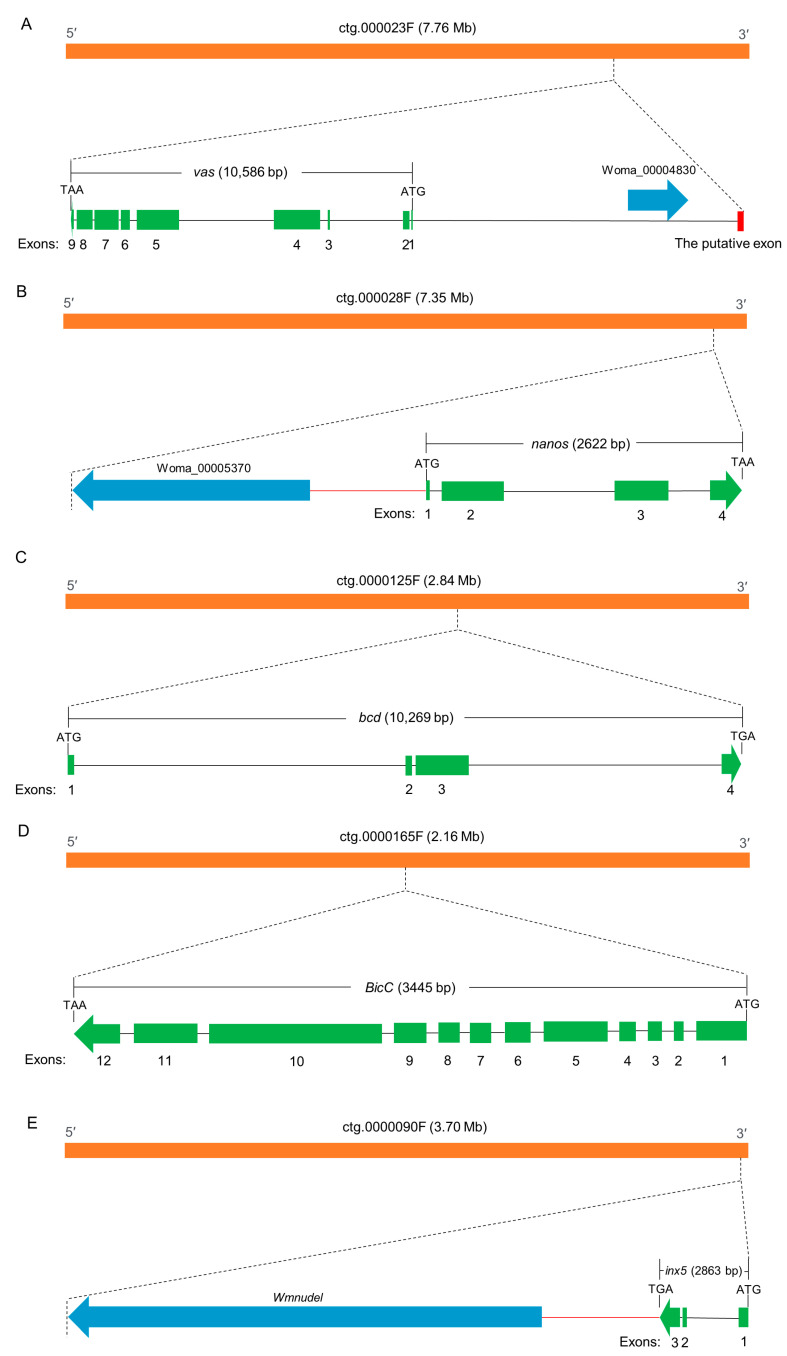
Diagrams of the genomic structures of *vas* (**A**), *nanos* (**B**), *bcd* (**C**), *BicC* (**D**), and *inx5* (**E**) in *W*. *magnifica*. The orange, green, blue, and red boxes represent contigs, exons, neighboring genes and the putative exon, respectively. The start and stop codons are marked. Arrows point in a 5′–3′ direction. The black horizontal line between the start codon and the stop codon indicates the introns. The red horizontal lines indicate intergenic regions, regulatory sequences, or/and untranslated regions. (Note: The *Wmvas* gene has two transcripts in the current genome annotation; in this figure, we only included the longest one).

**Figure 8:**
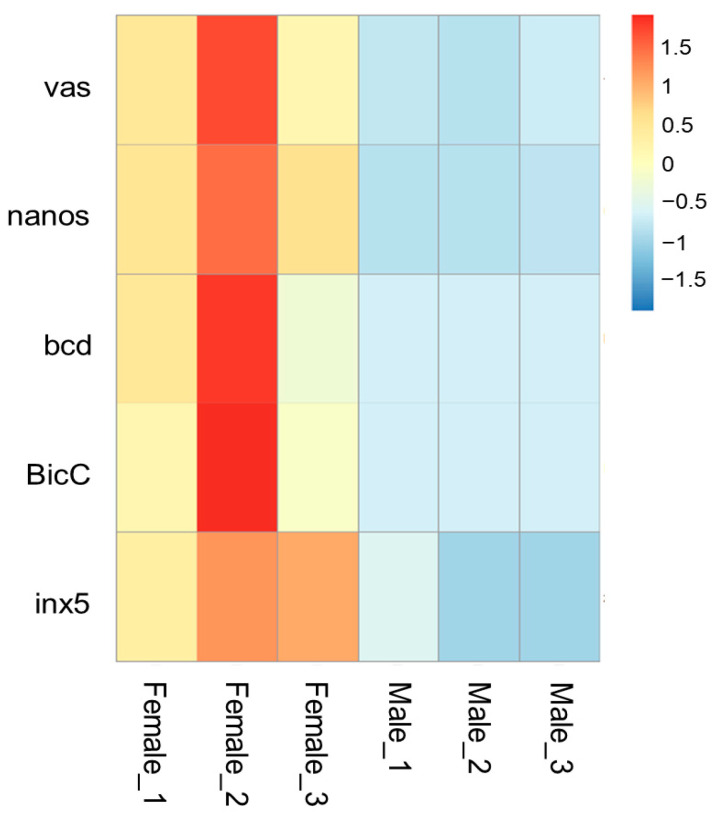
The heatmap of expression levels (RNA-seq analysis) of the *vas*, *nanos*, *bcd*, *BicC*, and *inx5* genes in females and males of *W. magnifica*. Rows represent genes and columns represent sample names. The red, yellow, and blue colors indicate high, medium, and low expressions, respectively.

*vas* (Woma_00004829) is localized at contig ctg.000023F. At approximately 6000 bp upstream of the start codon, we identified an ortholog of the *vasa intronic gene* (*vig*) of Drosophila, which was named SERPINE1 mRNA binding protein 1 (*Serbp1*, Woma_00004830) in *W. magnifica*. In *Drosophila*, the *vig* gene is located between the first non-coding exon and the downstream exon containing the start codon of the *vas* gene. Similarly, in *W. magnifica*, RNA-seq data supports that the *Wmvas* gene also initiates coding from the second exon, and the first non-coding exon does exist. This information is useful to accurately identify the sequence of the promoter. As the current annotation of the *W. magnifica* genome neglected the untranslated regions, the first non-coding exon of *Wmvas* is not annotated (Figure 7A). Similar to *C. hominivorax* and *L. sericata*, *Wmnanos* (Woma_00005371) contains four exons and the length of the coding region is 2622 bp. It is also linked to its upstream gene (Woma_00005370), the ortholog of CG11779 of *Drosophila* (Figure 7B). *bcd* (Woma_00011211) is present on contig ctg.000125F, and the organization is relatively simple with four exons (Figure 7C). *BicC* (Woma_00012414) is located in contig ctg.0000165F. *BicC* is relatively complicated with 12 exons (Figure 7D). Similar to other genes, *BicC* is only expressed in adult females, but *BicC* is not classified as a DEG. In *W. magnifica*, we identified an ortholog of zero population growth (*zpg*) (known as *inx4*), however, the expression levels of *Wmzpg* did not differ between adult males and adult females. In *C. hominivorax* [44] and *L. sericata* [50], no ortholog of *zpg* was found, and the *inx5* gene was considered to be an ortholog of *zpg*. Similarly, we also found an *inx5* gene (Woma_00010258) in contig ctg.000090F, which is very closely linked to *nudel* (Woma_00010257) and has only three exons (Figure 7E). The expression level analysis showed that all these genes, including *vas*, *nanos*, *bcd*, *BicC*, and *inx5*, were dominantly abundant in adult females (Figure 8 and Supplementary Materials Table S4).

**Figure 9:**
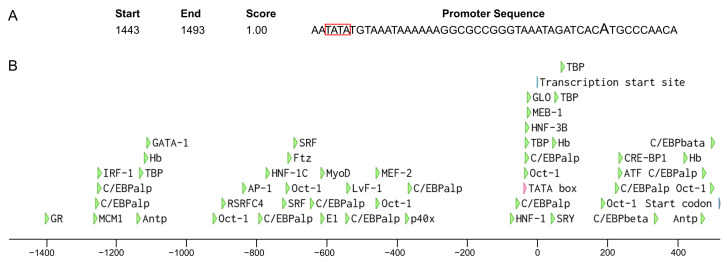
Analysis of the promoter of the *Wmnanos* gene. (**A**) The highlighted A indicates the predicted transcription start site. The TATA box is shown in the red box. Start and End represent the start and end of the sequence containing the transcription start site and the TATA box and the counting starts from the first position of the extracted 2000 bp sequence. Score represents the promoter score (between 0 and 1) (**B**) A schematic diagram of putative regulatory motifs in the 5′ flanking sequence. The colorful arrows represent the positions of transcription factor binding sites, the transcription start site or the start codon. The scale indicates the distance in nucleotides from the TSS at position 1.

We retrieved 2000 bp sequences upstream of the start codon of *Wmnanos* and performed a promoter analysis in silico. As a result, BDGP found a promoter sequence with a score of 1 containing the predicted transcription start site and TATA box (Figure 9A). Supported by AliBaba2.1, 49 transcription factor binding sites, such as for GATA binding protein (GATA-1), CCAAT/enhancer binding protein (C/EBP), activator protein-1 (AP-1), octamer-binding transcription factor-1 (Oct-1), Hunchback (Hb), TATA-binding protein (TBP), etc., were predicted (Figure 9B). 

Furthermore, we identified six U6 RNA genes with 96 bp, 111 bp, 104 bp, 99 bp, 96 bp, and 106 bp in five contigs of the *W. magnifica* genome. Two U6 genes were present in contig ctg.000034F, while the other four U6 genes were distributed in contigs ctg.000025F, ctg.000033F, ctg.000037F, and ctg.000437F (Supplementary Materials File S3).

### 3.5. Potential Target Genes for Control Strategies against W. magnifica

In *W. magnifica*, we inferred 972 genes, whose *D. melanogaster* orthologs are single-copy and associated with lethality and semi-lethality upon disruption (Supplementary Materials Table S5). These genes can be used for screening potential candidate targets for the development of vaccines, insecticides, or genetic control measures.

## 4. Discussion

### 4.1. Acquisition of Full-Length Sequences of Transcripts

We obtained the female transcript of *Wmtra*, and the non-sex-specific transcript of *Wmtra2* using the Iso-Seq technique. Unexpectedly, we were unable to obtain the male transcript of *Wmtra* from the Iso-Seq dataset. Combining RNA-seq and Iso-Seq data, however, we found that most of the genes captured by Iso-Seq have relatively high expression, indicating that the obstacle to identifying the male-specific transcript of *Wmtra* may have been an insufficient Iso-Seq sequencing depth. Therefore, with the aim of obtaining more thorough transcripts, especially those with low relative expression in insects, it may be necessary to conduct deeper sequencing. Usually, the full-length sequence of an RNA transcript can be obtained using a molecular biology approach known as rapid amplification of cDNA ends (RACE) if the sequence is only partially known. To date, many transcript sequences of *tra* or *tra2* in insects have been identified by the RACE method [48,49]. Iso-Seq represents an alternative method that enables the acquisition of full-length transcript sequences, including the entire coding sequence and untranslated regions. When combined with a technology that selects full-length capped and polyadenylated RNA molecules, the Iso-Seq method can maximize the repertoire of full-length transcripts for the objectives of the study. In comparison, the RACE approach only enables a limited number of target genes.

### 4.2. The Putative Sex Determination Mechanism of W. magnifica

The *tra and tra2* genes are pivotally important in insect sex determination. In the study, we isolated and characterized the two sex-determination genes in *W. magnifica*, *Wmtra* and *Wmtra2*, as well as their corresponding proteins, female WmTRA and WmTRA2. Similar to *L. cuprina* and *C. hominivorax*, *Wmtra* produces sex-specific transcripts and *Wmtra2* generates a single non-sex-specific transcript. We performed a multiple alignment of protein sequences between WmTRA/WmTRA2 and their orthologs in the myiasis-causing blow flies and the results indicate that TRA/TRA2 proteins are highly conserved among them. The phylogenetic analysis was in agreement with the taxonomic relationship, forming a cluster with proteins in blow flies.

In WmTRA, we also found four known TRA-specific domains [51,52,53] and two characteristic regions of the SR protein, including an RS domain and a proline-rich region at the C-terminal end [51,54]. The RS domain is found to mediate protein–protein interactions [54]. The second domain, TRACAM, was complete in the female WmTRA protein, but truncated in the male non-functional WmTRA protein due to the presence of the male-specific exon M1. In *M. domestica*, the molecular role of the TRACAM domain of MdTRA is in connection to the auto-regulatory function of *Mdtra* [55]. In *D. melanogaster*, the TRACAM domain is absent in non-auto-regulatory DmTRA. In contrast, a replaced *Sex-lethal* (*Sxl*) gene acts as an upstream regulator [52] instead of *tra*, suggesting the TRACAM domain may function in *Wmtra* auto-regulation. The other domains are found to be conserved in dipteran species, but the function remains unknown, such as the third domain (DIP domain) [51,55]. Furthermore, with a similar relative location to *L. cuprina* and *C. hominivorax*, we observed five TRA/TRA2 binding sites present in the male-specific exon and in the intron 1 of *Wmtra*, as well as two RS domains and an RNA-recognition motif in WmTRA2. These findings may indicate that WmTRA and WmTRA2 interact to form the TRA/TRA2 complex and bind to its own pre-mRNA, resulting in the auto-regulative splicing of *Wmtra* and the skipping of the male-specific exon. In non-Drosophilidae species, such as the medfly and the housefly, maternal deposition of *tra* mRNA in developing XX embryos translate into functional proteins and initiates the positive auto-regulatory loop of female-specific splicing, resulting in female differentiation [51,56]. In contrast, an M factor on the Y chromosome in XY embryos suppresses the *tra* function and, as a consequence, the initiation of the auto-regulatory loop is inhibited, promoting male development [56]. Future work investigating the expression pattern of *Wmtra* at different developmental stages, especially the early embryo, and the molecular function of the upstream Y-linked M factor will facilitate a better understanding of the sex determination mechanism in *W. magnifica*. Despite the successful identification of *tra* and *tra2* orthologs in *W. magnifica*, the investigation of gene function could not be pursued due to existing constraints on laboratory rearing of *W. magnifica*. Our next studies aim to employ advanced functional genomic tools, such as RNAi-mediated gene knockdown and CRISPR/Cas9-mediated gene knockout, for further investigation of their functions.

### 4.3. Genetic Controls against W. magnifica Infestation

Eradication of the New World screw-worm fly from the United States and later from Mexico and Central America through successive releases of radiation-sterilized flies produced at a mass-rearing facility demonstrates the effectiveness of SIT in insect pest control [25,26]. For SIT, however, females may consume half of the feed in a mass-rearing plant, although only males are effective in suppressing local populations. Eliminating females from the rearing process, therefore can result in significant savings in food costs. In a conditional female lethal transgenic strain developed for myiasis-causing blow flies by Concha et al. [28] and Yan et al. [29], only males survived in the absence of tetracycline. As an essential component to turn the sex-specific expression of a pro-apoptotic gene on or off in this system, the isolation of the *tra* gene plays an integral role in the development of the strain. In this study, we successfully identified and characterized *Wmtra*. Similar to other myiasis-causing flies, such as *L. cuprina*, *C. hominivorax*, *Wmtra* transcripts are spliced in a sex-specific manner, so that only the female transcript creates a full-length functional protein, whereas males encode, presumably, non-functional peptide. Therefore, by introducing the key first intron of the *Wmtra* gene to control the sex-specific expression of a pro-apoptotic gene in this system, we expect that the conditional female lethal transgenic system may work well in *W. magnifica*, as was the case for other myiasis-causing blow flies, *C. hominivorax* and *L. cuprina*, which has proven to be quite successful [28,29].

Gene drive systems promise to offer another powerful pest genetic control tool. Cas9-based homing gene drive systems have been developed in mosquitos [30,31,32,33,34]. For a successful gene drive system, the identification of precise sites in the target insect genome that are vital for female development, survival, or fecundity, is a key prerequisite. Furthermore, promoters from genes active in the germline to drive Cas9 expression, as well as promoters from U6 RNA for expression of gRNA are important. In the Medfly, *tra*-knockdown XY males develop normally, while XX individuals develop as fertile males [56]. Therefore, *Wmtra* can serve as such a target and female individuals are expected to be converted into males. Theoretically, this approach could result in an all-male population.

We analyzed DEGs between adult females and adult males and functional analysis showed that among upregulated genes those related to meiosis and mitosis GO terms were enriched, while among downregulated genes those related to muscle cell development and aerobic metabolic processes were enriched. These results likely correspond to the biology of the species, females invest in producing eggs, males in muscle for a mating flight. The female- and male-biased gene sets identified can also provide a useful resource for Cas9-based homing gene drive systems. For example, from the female-biased gene set, we identified several candidate genes, including *vas*, *nanos*, *bcd*, *BicC*, and *inx5*. Specifically, promoters from *nanos* and *vas* have been used in gene drive strains of mosquitoes to direct expression of the Cas9 nuclease [32]. In *D. suzukii*, promoters from either early germ cells, e.g., *vas* or *nanos*, or from late germ cells, e.g., *BicC*, have successfully driven the expression of the Cas9 nuclease [57]. Moreover, in the synthetic Medea toxin-antidote gene drive system, the promoter from *bcd* was employed to express a maternal toxin [58]. Among these, the *nanos* gene with a relatively simple organization should facilitate the isolation of the promoter. Also, the promoter from *nanos* was successfully applied in *D. melanogaster* and *D. suzukii* [59,60]. Therefore, a promoter from the *nanos* is a suitable candidate for driving Cas9 expression for further development of Cas9-based homing gene drives of *W. magnifica*. We investigated the promoter region of *Wmnanos* and identified 49 transcription factor binding sites. In the position of the TATA box, TBP binding sites are present, which may indicate that TBP binds to the TBP motif, facilitates the assembly of the pre-initiation complex, and promotes the recruitment of other transcription factors and RNA polymerase, ultimately leading to the initiation of transcription.

In the present investigation, our research findings revealed the maternal expression patterns in these genes in accordance with previous observations in other dipteran species. Nevertheless, due to the oviparous nature of *W. magnifica* and the current challenges in establishing comprehensive laboratory-rearing protocols, acquiring early-stage embryos directly from the field remains exceptionally arduous. In prospective investigations, we will undertake comprehensive examinations and refinement of laboratory-rearing protocols for *W. magnifica*, with the objective of detecting the gene expression patterns at early embryonic stages, facilitating the development of the genetically modified system in this species.

The promoter from the U6 RNA gene is ideal for driving gRNA transcription in Cas9-based homing gene drive systems. In different mosquito species, U6 regulatory sequences were employed to promote gRNA expression, with various degrees of activity [61]. In *W. magnifica*, the promoters from six U6 genes could be used to drive gRNA expression.

In summary, the identification of these genes can contribute to the genetic control of *W. magnifica*. In the conditional female lethal transgenic systems, the sex-specifically spliced *Wmtra* gene can be used to control the sex-specific expression of a pro-apoptotic gene. In the Cas9-based homing gene drive systems, *Wmtra* can serve as a target to convert females into males, and the promoters from *vas*, *nanos*, *bcd*, *BicC*, and *inx5*, as well as the promoters from U6 genes, can be applied to express Cas9 nuclease and gRNA in the germline, respectively. 

## 5. Conclusions

We successfully isolated and characterized the sex-determining genes *Wmtra* and *Wmtra2* in *W. magnifica*. *Wmtra* transcripts are sex-specifically spliced so that only the female transcript encodes a full-length functional protein, while the male transcript encodes a truncated and non-functional polypeptide due to the presence of the male-specific exon M1 containing multiple in-frame stop codons. The existence of five putative TRA/TRA2 binding sites in and around the male-specific exon M1 of *Wmtra* and the presence of an RNA-recognition motif in WmTRA2 may suggest that WmTRA interacts with its own pre-mRNA through WmTRA-2, resulting in the skipping of the male-specific exon M1. The comparative study showed that both the WmTRA and WmTRA2 proteins exhibited a high degree of similarity to their orthologs in the myiasis-causing blow flies, *L. sericata*, *L. cuprina*, and *C. hominivorax*. The sex transcriptome analysis reported 657 upregulated and 365 downregulated genes. Functional analysis showed that upregulated genes related to meiosis and mitosis were enriched, while downregulated genes were enriched in muscle cell development and aerobic metabolic processes. From the female-specific gene set, we identified five candidate genes, *vas*, *nanos*, *bcd*, *BicC*, and *inx5*, whose promoters can drive Cas9 expression in the germline in Cas9-based homing gene drive systems, as established in some dipteran species. The identification and characterization of these genes represent an important step in the development of genetic control programs for *W. magnifica* infestation.

## Data Availability

All transcriptome data were deposited at National Center for Biotechnology Information’s Sequence Read Archive (NCBI’s SRA) database (https://www.ncbi.nlm.nih.gov/sra, accessed on 8 March 2023) under the BioProject accession number PRJNA941182. The accession number for Iso-Seq data of the mixed different developmental stages and sexes is SRR23730896. The accession numbers for RNA-seq data of adult females and adult males are from SRR23731231 to SRR23731236.

## Supplementary Materials

The following supporting information can be downloaded at: https://www.mdpi.com/article/10.3390/insects14070620/s1, Figure S1: The PCA results of RNA-seq of three adult females and three adult males. File S1: Gene, transcript, and protein sequences of *Wmtra*. File S2: Gene, transcript, and protein sequences of *Wmtra2*. File S3: U6 RNA sequences in *W. magnifica*. Table S1: Statistics of RNA-seq data of adult females and adult males. Table S2: DEGs between adult females and adult males. Table S3: GO enrichment analysis of DEGs. Table S4: Expression levels of five female-biased genes. Table S5: *W. magnifica* orthologs of essential genes in *D. melanogaster*.
