# Supplementary material for: Genome and Transcriptome Analyses Facilitate Genetic Control of Wohlfahrtia magnifica, a Myiasis-Causing Flesh Fly
Source: Insects. 2023 Jul 10;14(7):620. doi: 10.3390/insects14070620 (PMC10380434; doi:10.3390/insects14070620)
Supplement: Supplementary file 1 [file insects-14-00620-s001.zip › File_S1.pdf]

>ID=Woma_00009986; type=gene; loc=ctg.000086F:complement(833397..837790); name=*Wmtra*;
length=4394

GGCGTCAAGTTGATTAAGAGTGTAGCAAAATTTTCGGGAAGGTTAGGCTGTAGCTAAAAG
TACAAATTGGATAAAATTGGAGACCTTGGCGACATTGTTGTTTTTATTACAAAACAAATA
AATATTTAGTTTTTTGGGCCAAAAGGAAAGTTTCGCCTGTTACCATCAACACCATCATCT
ATCGCCTTCTCAACTTTATTACGACCACAGCAACAATTAGAAATGGATTCCATAGCATCT
GGCTTAGCAACATCAGCTCTAAATATACCAAAGATTAAAATTCATCAATCTATACCAAGT
GATTCCATAAGAAGAGGTCCCCGTGCCATCGTACGCTCAGCTGCTTTAAACGACGACATT
AATATTTTACGCAGATTTGGTAATTTTCTTACAAAATTTCTGCATACCTTTTAAATTTTT
TTGCAGTATTTAATACAATGAGAGTAGTTACAAAGAAATTAACAACATTATAAGGAACGT
AAAAGAAATTAGATATTTTTATCAGTAATTATTGAATGGCACAATGAAAGAAAAAAGCTA
CTATTGAAGGCTGTGACGCGATATGCAGCGCTAGAAAAATTCATCAATCAACATTCCATA
TCCATTTCCTTGAACAGTGAGGAGAATCTTCAAACAACTAAACCGCTGACTACAACTGTG
AGTATTACAATTACATTTTTATTATATTAAAAAAAAAAAAACAATTTTCTAATGGTTCTAA
GTGTTAACAATCGTTTTTCTTTTGTATATATTTCAAAAAATTCATTTTGAAACAATCGCC
TCAATGGTACACTCATACCTCCTTATACACCCTTTGTTTTTATGGCATTTCGCTATAACG
GCTTGTCTAAATTGTGGAACATCTTCGCTTTACGGCACAAAATACCCCATCATACGGTAA
AAAAGACAGTATTTCATCGTTTTTAAGGAATAGTGTAAGACAAGCGAAATCAACGCGCTT
TACGGCATTTTAATTTACGGCCTACATTTTCAGAGCCTAACTATGCCGCATAAAGAGGTG
TGAGTGCATTAAAAACAACCATTGTTGCTAGTGTCTATCGGGGTGGCATTTTTACTCTAT
ACACCGATAAAGCTAATGTTATGGTATACATATGTCCAGCTATACCCTAGTTTTTTTGTA
TTCACTACTTATTATCTTATTCTTAGTAATGCTGTGAAATACTTTTTAATTCTTTGTTCG
TCACTAAAGACAGTAATTACGTAGTAATTGATTTACTAAAATAATTGCTCTATCATTTGG
TAACTAGCACTCCTACTGCAATTTCGTAGTAAGCAGGAACCTGTAAACAACACTGCAAGC
AGGTAAAGGAGATGGGTGAATCACCACCTTGTAAGACATCCCCATGTTTATTTTGAAAAC
ACACAAAACAATTATAACTATAGATAGATAAAATTGTTCTAAGTTTGATACATATATATT
TTTTTTAATTTGAAAAATTTTTTGATCTCGAGTCATTAATATGTAAATGCTAACAACGCT
GATCTCGAAGTTAAAAGCAAATGTTTACTTTTCACATTAGACTAATTTTATAATACGCTC
TGCAGTCTTCTATTTTGACATTTATATAAGTAAAATTAAATCATTTCAGAAACAATTTCT
TTAGCGAAATCCGAATATAGTTTTGCCGTCATATAAAAGCCAAGTAACACTTATTAAGCT
TTCTAATCTTAAGAACAGAATTACTTCCACAAACATTTGTTTTTTAATAATCTGATATGC
GATATTATTGGTTCTGTGGACCTTCAGATTAAGTTAGAATTTATTACTCCAATTAGTCCT
TTAAAGTGCAATATTTTCAGTCCAATTACGCAAATTTTTATATATTCCAAAGAATGTATA
CACGATTTGACATTTTAGTTTTAATAACTAGTATAGCAATTTTAAAAGTAGAATTATTGA
TTAAATATGTATTAAAGTTATATTTTATTTAAATGTGTATTGAAATAGTATTATTAATAT
AGCTTGTAAAAATTAATTTGCAAGCTTAACAATAATTTTAAAAATCAATAAATCCAAATA
TTTATACTTATATGGTTATAGTTATTTTATTAAAATAAAAATAAAATGTTAACTTATAAA
TTTGTTAGGATAGATAAGTGCAATTAATTTCAGAGTAGAGATTTATCAACCATAACAAAA
ATCTGCAATCAACATACCATATTTCAAAGACGATGGGAAATATCTACATACGTCAATCAA
CATACAATTTGGAGAGTGTTTTCTAAATATACATTAAATTCTACAATCAACATACCAACT
ACATGGCAATATTTCAATCAACTATAAAAATTATCAACAATCAACATTCCAAAATTGGAC
AATGATGATAAAATTTTAAAAATGTTACATCTGAGCAATATGTAATTTTTCTAAACAATA
AAATTATGTATATAATTTATAAATGTCGAAATCAAATATAAATTAAAATTTTAAAAATAT
TGTTCCTCTAAAAAACGGTTACACTTGCCATAACGACTTGAATTAATATTGTAAAAAATA

ATTTAACTTCACAAAAATCTGTAAATTTATACTTTTCAAAATCTTGCCGCAATGCAACAT
ACTACTAAATTATATATTTTAAATTAAGAAATATTCATGGATATTTATCAACACTCAATG
CCTAAGTCAAACTCATTTCTGTATATGATAAAATTTAATTGTTTTTCTCATGTTTAGGTG
AAGGTTCTAGACCGTTATTTGAACGTGATGATATAACTGTCAATCAGCCCACTGTAGGAG
ATTCACTCGAGAATATAACAAGCCATGATTGTAAAAATGGTTGTAAAGCTAAAAATTATA
AAAGTTCGTCAATTGTAAGTTGTGCCATTTAAGTGTTATAAGTACATAATAATGCTCTCT
GTATATTAAGTTTATATTAATTTCCTTTGAAATATAAACAATTTGAAATTTAATTCATTT
GTATAAAGTAGAAAATATGATCTAAAATTTACTTTTATACAAGTCAAATAGAAACAAAAG
ATATTAATTGGATTATTTCAAAGGAATTTAATAAAACGTAAAACATGAGCTACAATTATT
CTTACAAAACTTTTTTTAATATAACTGCAAAGGAATCGAGTTCACCTGAAAGATACCGAC
ATCGAGATGCCAAAAAACGTTCTTCTCCACCCTCATCTGCGCGACGCAAGTCACCTGAAA
AATCTAGTCGTGATGAAAATTCGAGTCATAGTCATAATAGAAAGTACAATGAATATATAA
AAAATAAGAGTTGCAATACAAAGACGACTGAAAGATGTGGTAGAAGACGAGAAAAAAGCA
AATCTCGTACACCATCGTCTACAAGAATAAAGACATTAGCAGAGAAAAAACCATATTACA
GGGATGAACAACGCGAAAGAGATCGTAGAAGACGTTTATATGGACATTCACGTTCTCGTA
CACCTCCAATAATTTCAAGTAGAAGGTATAGCCCACACTTATCTAGAAGGCGTGTTGGCA
GCCATACGAGAAGAAGGCGTTCATCTAGCAGAGATATAAATTATTCCCCTTATCATAGTT
TCATGAGATATCATAGAAGTCGAAGATCTCGAACTCGTTCAAAAAGCCGAACACCATCCT
CAAGAAGAGACCGAGAACGTCACAAATCCCATTATAATAGAGAAAGACATAAAGAACAAA
AGGATAATTCAAATGCTTTAACAACTCCAGTTATTGCTGCTCCACCACCACAGATCATTC
CAATAGCAGTTCCTGTACCGGCGGACTATGCTTATACATTTGTATGTTGCTAATTTATAG
CAATAAATTTTTAGTATATAGCTTTTAATTTGTAAAATTATATCATTTTAGCCTGGTTGG
ACGGCAACTCCGCAGGCATCCTGGCATACACCTCATCATCGGCCACCTACAGCTCCTACT
CATTATGCTCCATTTCCTATGCTGCCAATGATGCCTCCACTTAGAGCTGCACATCGGACT
CCATTTGGTGGTTTGCCGCCTCCATTACCTTATGGTCCAATGCCGCCTGGCTATCGGCCG
CACACTGGTCCAAGATATAATACCCCTAGGCATAATAACACACACAACTCGCATGCTCGT
CCTAAAAAACCTACAAACTAGAGAGGTGTTGTATGCATATTAATAAGCAATCAATGTATA
TATAATTAGTTTTGATTTAAAACTTTTGTATGGATGTTTAAAAATCTTTGTCGAAATGGA
AGAGTGGCATATATTTATATGATATAAGACAAGTTACGAAAAAGGAACGTCTAAAACGTT
TTCTTTTTTTTCAATATACGTGAACTCTGTAATTTTTTTCCAGATTGTCTCTAAATATGA
TAAATCTGTGGTCATGTGATAGAATAATTTAAGCTACTCTTCCATATAGACACAGAAATA
AAAAAATTTTGGCA
>Parent=Woma_00009986; type=transcript;
loc=ctg.000086F:complement(join(833397..833959,834030..834698, 834957..835093, 837412..837790));
name=female-specific *Wmtra* transcript; length=1748
GGCGTCAAGTTGATTAAGAGTGTAGCAAAATTTTCGGGAAGGTTAGGCTGTAGCTAAAAG
TACAAATTGGATAAAATTGGAGACCTTGGCGACATTGTTGTTTTTATTACAAAACAAATA
AATATTTAGTTTTTTGGGCCAAAAGGAAAGTTTCGCCTGTTACCATCAACACCATCATCT
ATCGCCTTCTCAACTTTATTACGACCACAGCAACAATTAGAAATGGATTCCATAGCATCT
GGCTTAGCAACATCAGCTCTAAATATACCAAAGATTAAAATTCATCAATCTATACCAAGT
GATTCCATAAGAAGAGGTCCCCGTGCCATCGTACGCTCAGCTGCTTTAAACGACGACATT
AATATTTTACGCAGATTTGGTGAAGGTTCTAGACCGTTATTTGAACGTGATGATATAACT
GTCAATCAGCCCACTGTAGGAGATTCACTCGAGAATATAACAAGCCATGATTGTAAAAAT
GGTTGTAAAGCTAAAAATTATAAAAGTTCGTCAATTGAATCGAGTTCACCTGAAAGATAC

CGACATCGAGATGCCAAAAAACGTTCTTCTCCACCCTCATCTGCGCGACGCAAGTCACCT
GAAAAATCTAGTCGTGATGAAAATTCGAGTCATAGTCATAATAGAAAGTACAATGAATAT
ATAAAAAATAAGAGTTGCAATACAAAGACGACTGAAAGATGTGGTAGAAGACGAGAAAAA
AGCAAATCTCGTACACCATCGTCTACAAGAATAAAGACATTAGCAGAGAAAAAACCATAT
TACAGGGATGAACAACGCGAAAGAGATCGTAGAAGACGTTTATATGGACATTCACGTTCT
CGTACACCTCCAATAATTTCAAGTAGAAGGTATAGCCCACACTTATCTAGAAGGCGTGTT
GGCAGCCATACGAGAAGAAGGCGTTCATCTAGCAGAGATATAAATTATTCCCCTTATCAT
AGTTTCATGAGATATCATAGAAGTCGAAGATCTCGAACTCGTTCAAAAAGCCGAACACCA
TCCTCAAGAAGAGACCGAGAACGTCACAAATCCCATTATAATAGAGAAAGACATAAAGAA
CAAAAGGATAATTCAAATGCTTTAACAACTCCAGTTATTGCTGCTCCACCACCACAGATC
ATTCCAATAGCAGTTCCTGTACCGGCGGACTATGCTTATACATTTCCTGGTTGGACGGCA
ACTCCGCAGGCATCCTGGCATACACCTCATCATCGGCCACCTACAGCTCCTACTCATTAT
GCTCCATTTCCTATGCTGCCAATGATGCCTCCACTTAGAGCTGCACATCGGACTCCATTT
GGTGGTTTGCCGCCTCCATTACCTTATGGTCCAATGCCGCCTGGCTATCGGCCGCACACT
GGTCCAAGATATAATACCCCTAGGCATAATAACACACACAACTCGCATGCTCGTCCTAAA
AAACCTACAAACTAGAGAGGTGTTGTATGCATATTAATAAGCAATCAATGTATATATAAT
TAGTTTTGATTTAAAACTTTTGTATGGATGTTTAAAAATCTTTGTCGAAATGGAAGAGTG
GCATATATTTATATGATATAAGACAAGTTACGAAAAAGGAACGTCTAAAACGTTTTCTTT
TTTTTCAATATACGTGAACTCTGTAATTTTTTTCCAGATTGTCTCTAAATATGATAAATC
TGTGGTCATGTGATAGAATAATTTAAGCTACTCTTCCATATAGACACAGAAATAAAAAAA
TTTTGGCA
>Parent=Woma_00009986; type=protein;
loc=ctg.000086F:complement(join(833690..833959,834030..834698, 834957..835093, 837412..837568));
name=female-specific WmTRA protein; length=410
MDSIASGLATSALNIPKIKIHQSIPSDSIRRGPRAIVRSAALNDDINILR
RFGEGSRPLFERDDITVNQPTVGDSLENITSHDCKNGCKAKNYKSSSIES
SSPERYRHRDAKKRSSPPSSARRKSPEKSSRDENSSHSHNRKYNEYIKNK
SCNTKTTERCGRRREKSKSRTPSSTRIKTLAEKKPYYRDEQRERDRRRRL
YGHSRSRTPPIISSRRYSPHLSRRRVGSHTRRRRSSSRDINYSPYHSFMR
YHRSRRSRTRSKSRTPSSRRDRERHKSHYNRERHKEQKDNSNALTTPVIA
APPPQIIPIAVPVPADYAYTFPGWTATPQASWHTPHHRPPTAPTHYAPFP
MLPMMPPLRAAHRTPFGGLPPPLPYGPMPPGYRPHTGPRYNTPRHNNTHN
SHARPKKPTN
>Parent=Woma_00009986; type= transcript;
loc=ctg.000086F:complement(join(833397..833959,834030..834698, 834957..835093, 837134..837790));
name=male-specific *Wmtra* transcript; length=2026
GGCGTCAAGTTGATTAAGAGTGTAGCAAAATTTTCGGGAAGGTTAGGCTGTAGCTAAAAG
TACAAATTGGATAAAATTGGAGACCTTGGCGACATTGTTGTTTTTATTACAAAACAAATA
AATATTTAGTTTTTTGGGCCAAAAGGAAAGTTTCGCCTGTTACCATCAACACCATCATCT
ATCGCCTTCTCAACTTTATTACGACCACAGCAACAATTAGAAATGGATTCCATAGCATCT
GGCTTAGCAACATCAGCTCTAAATATACCAAAGATTAAAATTCATCAATCTATACCAAGT
GATTCCATAAGAAGAGGTCCCCGTGCCATCGTACGCTCAGCTGCTTTAAACGACGACATT
AATATTTTACGCAGATTTGGTAATTTTCTTACAAAATTTCTGCATACCTTTTAAATTTTT
TTGCAGTATTTAATACAATGAGAGTAGTTACAAAGAAATTAACAACATTATAAGGAACGT

AAAAGAAATTAGATATTTTTATCAGTAATTATTGAATGGCACAATGAAAGAAAAAAGCTA
CTATTGAAGGCTGTGACGCGATATGCAGCGCTAGAAAAATTCATCAATCAACATTCCATA
TCCATTTCCTTGAACAGTGAGGAGAATCTTCAAACAACTAAACCGCTGACTACAACTGTG
AAGGTTCTAGACCGTTATTTGAACGTGATGATATAACTGTCAATCAGCCCACTGTAGGAG
ATTCACTCGAGAATATAACAAGCCATGATTGTAAAAATGGTTGTAAAGCTAAAAATTATA
AAAGTTCGTCAATTGAATCGAGTTCACCTGAAAGATACCGACATCGAGATGCCAAAAAAC
GTTCTTCTCCACCCTCATCTGCGCGACGCAAGTCACCTGAAAAATCTAGTCGTGATGAAA
ATTCGAGTCATAGTCATAATAGAAAGTACAATGAATATATAAAAAATAAGAGTTGCAATA
CAAAGACGACTGAAAGATGTGGTAGAAGACGAGAAAAAAGCAAATCTCGTACACCATCGT
CTACAAGAATAAAGACATTAGCAGAGAAAAAACCATATTACAGGGATGAACAACGCGAAA
GAGATCGTAGAAGACGTTTATATGGACATTCACGTTCTCGTACACCTCCAATAATTTCAA
GTAGAAGGTATAGCCCACACTTATCTAGAAGGCGTGTTGGCAGCCATACGAGAAGAAGGC
GTTCATCTAGCAGAGATATAAATTATTCCCCTTATCATAGTTTCATGAGATATCATAGAA
GTCGAAGATCTCGAACTCGTTCAAAAAGCCGAACACCATCCTCAAGAAGAGACCGAGAAC
GTCACAAATCCCATTATAATAGAGAAAGACATAAAGAACAAAAGGATAATTCAAATGCTT
TAACAACTCCAGTTATTGCTGCTCCACCACCACAGATCATTCCAATAGCAGTTCCTGTAC
CGGCGGACTATGCTTATACATTTCCTGGTTGGACGGCAACTCCGCAGGCATCCTGGCATA
CACCTCATCATCGGCCACCTACAGCTCCTACTCATTATGCTCCATTTCCTATGCTGCCAA
TGATGCCTCCACTTAGAGCTGCACATCGGACTCCATTTGGTGGTTTGCCGCCTCCATTAC
CTTATGGTCCAATGCCGCCTGGCTATCGGCCGCACACTGGTCCAAGATATAATACCCCTA
GGCATAATAACACACACAACTCGCATGCTCGTCCTAAAAAACCTACAAACTAGAGAGGTG
TTGTATGCATATTAATAAGCAATCAATGTATATATAATTAGTTTTGATTTAAAACTTTTG
TATGGATGTTTAAAAATCTTTGTCGAAATGGAAGAGTGGCATATATTTATATGATATAAG
ACAAGTTACGAAAAAGGAACGTCTAAAACGTTTTCTTTTTTTTCAATATACGTGAACTCT
GTAATTTTTTTCCAGATTGTCTCTAAATATGATAAATCTGTGGTCATGTGATAGAATAAT
TTAAGCTACTCTTCCATATAGACACAGAAATAAAAAAATTTTGGCA
>Parent=Woma_00009986; type=protein; loc=ctg.000086F:complement(837377..837568); name=male-specific WmTRA protein; length=63
MDSIASGLATSALNIPKIKIHQSIPSDSIRRGPRAIVRSAALNDDINILR
RFGNFLTKFLHTF
