# Supplementary material for: Genome and Transcriptome Analyses Facilitate Genetic Control of Wohlfahrtia magnifica, a Myiasis-Causing Flesh Fly
Source: Insects. 2023 Jul 10;14(7):620. doi: 10.3390/insects14070620 (PMC10380434; doi:10.3390/insects14070620)
Supplement: Supplementary file 1 [file insects-14-00620-s001.zip › File_S2.pdf]

> ID=Woma_00008264; type=gene; loc=ctg.000059F:3139052..3142296; name=*Wmtra2*; length=3116
ATAGACGGCTTTGCTTTTGTACAGTGTTTTGATAAATTTCTATTTTGTTTAAATCAAATA
TTTTTTTTGGCGAAATTGAACATTTACGGAGGAAAAATTAAGATTTATTATAATTTAAAC
AAACTAAGCTTTATATTAAGAGTTACAGAACAAAATGGTAAATTATTTCCCCTGGATACT
TTCTCATCAAAAATCATTTGATTAATTATAATTTAAAGCATTTATGTATTCAATTTACAT
ATTTTTCTTGTACTATTTTCAGAGTCCTCGTTCACGCAGTCGTTCCATAACACCACGTCG
TTCATATTCTCGCTCACCCTATAGACGCAGCGGACGCCGGTAAGTAGTGTAAAATTGCCC
TTTAGTTATTGGTTATATTTGATTACAATTTTCTTCTCTGATTGTATAAATGAAGAAAAT
CTTACAGCCGTCATAAGTATGACTCTCGTAGTAGTTCCAGTTCGCATCCGCCGTCGCCAC
CACCACTTACATCTAGGCGACACAATAGCCGTTATTCCAGCCGCAGTATATCAAGGTAAG
TTCTGCTTAATTTGCATCAAATTTAGAGGTTAGGTTGAGCACATTTGAAAATTTTAAAAA
AATTTTTATTAATTGAATAGCTTGGATAAAATAATTCTAAATGTGTATATATATATATAT
ATATATATATATATATATATATATATATATAAAAAACATATATATATATATATATATATA
TATATATATATATATATATATATATATATATATATATATATATATATATATATATATATA
TATATATATATATATGTTTTTTGAAAACTGCCTTTTTATGGTTTTCTTAAAGTAGATATA
AATAATGGATGGATTTTTAACGCCTGTCTATTTTTAAAACTTAAAATATTTTAAGTTTGG
TTAAATAATGCAGGTACTTTTATAGCTTAAGAATTAATTTCAGAGAAAGCTAATTTAACT
CGTATTTTCTTTATTATTTAGTTCAGGGCTTAAAATAATTGTTTCATTCAAAATATTTCA
TAAAGTTATCATAATATTGTATATAGTATGACTCATTTTATTATATAAAATTGTAACATA
TTGATAATTTATTTTTAGGAATTTGAATTCTGAATTTTTTCTATTCAAATCAAACTATTG
AAATAATTAAAGTTTTTAGCAAATTTCAACTAAACAACATGCAGCAATTAAAATGGCAAA
CTCAAAATAATATATAGAAACGATGCAAGTAACTTCAAATACTTTCAGGTTTTCTTTGTT
TTTCAAAAACTAATCTAAAATATTGAAAAATCAAAGTGTAGCAAATTTCCATTACAAAAC
ATAACAATTATGGTACAAGAAAACCATACAACTTGGCAAAATTCTTTGAAAAACTAAGGA
TTTTTGTCAAATCAATGAAACTTAATTTGGAAATAATTCGAAGAATAATGCAAAACAAAA
AACGAACATATTGAAATAAACTTGAATATAAATCGTATTCGAAATACTAACGTTAATAAT
TTCTTTTTCGTTTTGTGTAATATTAAAATTTTTATTATTTTAATCCTGCAGATCCATATC
ACCCGATTATAGAAAACGTTATACTAGTCGTAGGCATTATTCGAAATCTCGTTCGAAAAC
CAAAAGCCGTTCAAGATCACGTTCACGCACATCGTACGATAAAAATATACGCAATAATAG
AGTAAGTAAATTGGAATATACATTTGAATTTTTATAATTTAACCAATCTAAAAAATTGAA
TTGAAATTGAGAGTTGAAGCAGTTTGTAAAAGTTTAGTTTAATTATATACTTTTAGGAAA
AACCTTTGCCTTGCCGTTGTATTGGAGTATTTGGTTTAAGCGTTTATACTACACAACAAA
AAATAAGAGAAATATTTTCAAAATTTGGTCCTATCGAACGTATACAAGTAATAACTGATG
CGCAGGTAAGGTTTATTTGCAATATTTTTGTCCATTTAATTAAAAGCCTTTTATTTTATT
CATTTTATTTAAAGTTGTAAGAAATAAAATGACACTCGAAGAGGCTAAAATAATTATTTT
GAACAATAATAAATAGAGTTTTGATTTAAGAATATTATAAACAAAACCAAAGGCTTGTTG
TTACACTATAAACGTAGTAACGTCGTTTTTTCAATACTGTTTTTATAGTTTTTTGTACTG
TTTTTTTATTTTATTTTTTTCATAGACCCGTCGTTCGCGTGGTTTTTGTTTTATTTATTA
TGATAATTTAGCTGATGCTAGAGCCGCCTGTGATAACTGTTGTGGCATGGAAATTGATGG
CAGACGTATACGTGTTGCCTATTCCATAACTGAAAGGCCACACACACCCACTCCTGGTGT
TTATATGGGAAAACCAGCAAAGTAAATATTCAATATTGAAATTTTTACATTTAAAATATT
GTTTTGTTGCATTTTTAGAGATTTAAGAGAACGTTATCGTGCCCAAAAGCAACAGCGCCA
TCATTCTCCAATTACTTATAAAAGTCACCGTAGTAGTAACCGCCATCATTATGAACGCAG
TCGTAGCCGTTCCTATACACCACGTAAGTAATGTTTTCATTATTTTCTATTACAATTTCT

TATACCTCAGGAGCGTTGTTAAGTTGTTGCGCCATTTTAATTTTAACTCCTTATTTATAT
GCTTTATGCTTTTGCTGCTACATTTTTGATTTGTAAATCATTTCAAAACATAGAATTGAG
TGTAAAGAATGAATTCAAAATATTAATTTTTAATATCTTTTACAGGTCGTTATCGTTACT
GATGCCCCTTGAAGTGTGTCAGTATTTATTCCATCGAACCATATGCATAATTTTTGAATG
CTCTTAATTTTTGAATAGTTTCTCTTTTTTACTTTTGCTTGTGTATTTTCATCATTTCAT
TTGTTTGTTTTTTTTTTATTATTTATTGGTATCGAGATTGAATTTTTTTCTGTTTTATGA
TTTATTGTTTAGTTTTAATTGTAACATAGAATATATTGAAAATATCATTTTTTCTGTTTG
AATACAGATTTCCTTAATTTTCTGGTAAATTTTTGTTTTATAAAAATTAATGAAATACAA
CAAAAAAGAGAAGCATTTCGTTTAAGAACGAATGCTTCTATTTATTTCCTTAACAT

>Parent=Woma_00008264; type= transcripts; loc=ctg.000059F:join(3139052..3139208,3139314..
3139390,3139467..3139586,3140603..3140732,3140848..3140976,3141237..3141412,3141470..3141594,314
1797..3142167); name=*Wmtra2* transcripts; length=1285

ATAGACGGCTTTGCTTTTGTACAGTGTTTTGATAAATTTCTATTTTGTTTAAATCAAATA
TTTTTTTTGGCGAAATTGAACATTTACGGAGGAAAAATTAAGATTTATTATAATTTAAAC
AAACTAAGCTTTATATTAAGAGTTACAGAACAAAATGAGTCCTCGTTCACGCAGTCGTTC
CATAACACCACGTCGTTCATATTCTCGCTCACCCTATAGACGCAGCGGACGCCGAAAATC
TTACAGCCGTCATAAGTATGACTCTCGTAGTAGTTCCAGTTCGCATCCGCCGTCGCCACC
ACCACTTACATCTAGGCGACACAATAGCCGTTATTCCAGCCGCAGTATATCAAGATCCAT
ATCACCCGATTATAGAAAACGTTATACTAGTCGTAGGCATTATTCGAAATCTCGTTCGAA
AACCAAAAGCCGTTCAAGATCACGTTCACGCACATCGTACGATAAAAATATACGCAATAA
TAGAGAAAAACCTTTGCCTTGCCGTTGTATTGGAGTATTTGGTTTAAGCGTTTATACTAC
ACAACAAAAAATAAGAGAAATATTTTCAAAATTTGGTCCTATCGAACGTATACAAGTAAT
AACTGATGCGCAGACCCGTCGTTCGCGTGGTTTTTGTTTTATTTATTATGATAATTTAGC
TGATGCTAGAGCCGCCTGTGATAACTGTTGTGGCATGGAAATTGATGGCAGACGTATACG
TGTTGCCTATTCCATAACTGAAAGGCCACACACACCCACTCCTGGTGTTTATATGGGAAA
ACCAGCAAAAGATTTAAGAGAACGTTATCGTGCCCAAAAGCAACAGCGCCATCATTCTCC
AATTACTTATAAAAGTCACCGTAGTAGTAACCGCCATCATTATGAACGCAGTCGTAGCCG
TTCCTATACACCACGTCGTTATCGTTACTGATGCCCCTTGAAGTGTGTCAGTATTTATTC
CATCGAACCATATGCATAATTTTTGAATGCTCTTAATTTTTGAATAGTTTCTCTTTTTTA
CTTTTGCTTGTGTATTTTCATCATTTCATTTGTTTGTTTTTTTTTTATTATTTATTGGTA
TCGAGATTGAATTTTTTTCTGTTTTATGATTTATTGTTTAGTTTTAATTGTAACATAGAA
TATATTGAAAATATCATTTTTTCTGTTTGAATACAGATTTCCTTAATTTTCTGGTAAATT
TTTGTTTTATAAAAATTAATGAAATACAACAAAAAAGAGAAGCATTTCGTTTAAGAACGA
ATGCTTCTATTTATTTCCTTAACAT

>Parent= Woma_00008264; type=protein; loc=ctg.000059F:join(3139206..3139208,3139314..
3139390,3139467..3139586,3140603..3140732,3140848..3140976,3141237..3141412,3141470..3141594,314
1797.. 3141813); name=WmTRA2 protein; length=258

MSPRSRSRSITPRRSYSRSPYRRSGRRKSYSRHKYDSRSSSSSHPPSPPP
LTSRRHNSRYSSRSISRSISPDYRKRYTSRRHYSKSRSKTKSRSRSRSRT
SYDKNIRNNREKPLPCRCIGVFGLSVYTTQQKIREIFSKFGPIERIQVIT
DAQTRRSRGFCFIYYDNLADARAACDNCCGMEIDGRRIRVAYSITERPHT
PTPGVYMGKPAKDLRERYRAQKQQRHHSPITYKSHRSSNRHHYERSRSRS
YTPRRYRY
