# Supplementary material for: Genome and Transcriptome Analyses Facilitate Genetic Control of Wohlfahrtia magnifica, a Myiasis-Causing Flesh Fly
Source: Insects. 2023 Jul 10;14(7):620. doi: 10.3390/insects14070620 (PMC10380434; doi:10.3390/insects14070620)
Supplement: Supplementary file 1 [file insects-14-00620-s001.zip › File_S3.pdf]

> U6 spliceosomal RNA; Loc=ctg.000025F: 1221473..1221568
GTTCTTGCTTCGGCAGAACATATACTAAAATGGGAACGATACAGAGAAGATTAGCATGGCCCCT
ACACGCAAAATCGTGAAGCGTTCCACATTTTT
> U6 spliceosomal RNA; Loc=ctg.000033F: 5094703..5094813
GTTCTTGCTTCGGTAGAACATATATATACTAAAATTGGAACGATACAGAGAAGATTAGCATGGC
CTTTGGGCACGGGTGACACGCAAAATCGTAAAGCCTTCCACTTTTTT
> U6 spliceosomal RNA; Loc=ctg.000034F: 542616..542719
AATGTCAATCCGACCTCGTACACTAAAATTGGAACGATACAGAGAAGATTAGCATGGCCCTGCG
TAAGTATGACTCGCAAAATCGTGAAGTGTGCCACATGTTT
> U6 spliceosomal RNA; Loc=ctg.000034F: 2328939..2329037
GCTCTTGCTTCGGCAGAACATATACTAAAATTGGAACGATACAGAGAATATTAGCATGGCTCTG
CGTAAGGATGACACGTAAAATCGTTCCACATTTTT
>U6 spliceosomal RNA; Loc=ctg.000037F: 2976518..2976613 reverse complement
GTTTTTCTGGCTTCGGTAGAACATACACTAAAATTGGAAGATACAGAGAAGATTAGCAAGGATG
ACACGCAAAATTTTGAAGCGTTCCACATTTTT
>U6 spliceosomal RNA; Loc=ctg.000437F: 403325..403430 reverse complement
CTTCTTGCTTCGGCAGAACATATACTAAAATTGGAACGATACAGAGAAGATCAGCATGGCCCTG
TGCAAGGATGACACGCAAAATCGTGAAGCGTTCCACATTTTT
