# Supplementary figures and images for: Genome and Transcriptome Analyses Facilitate Genetic Control of Wohlfahrtia magnifica, a Myiasis-Causing Flesh Fly
Source: Insects. 2023 Jul 10;14(7):620. doi: 10.3390/insects14070620 (PMC10380434; doi:10.3390/insects14070620)

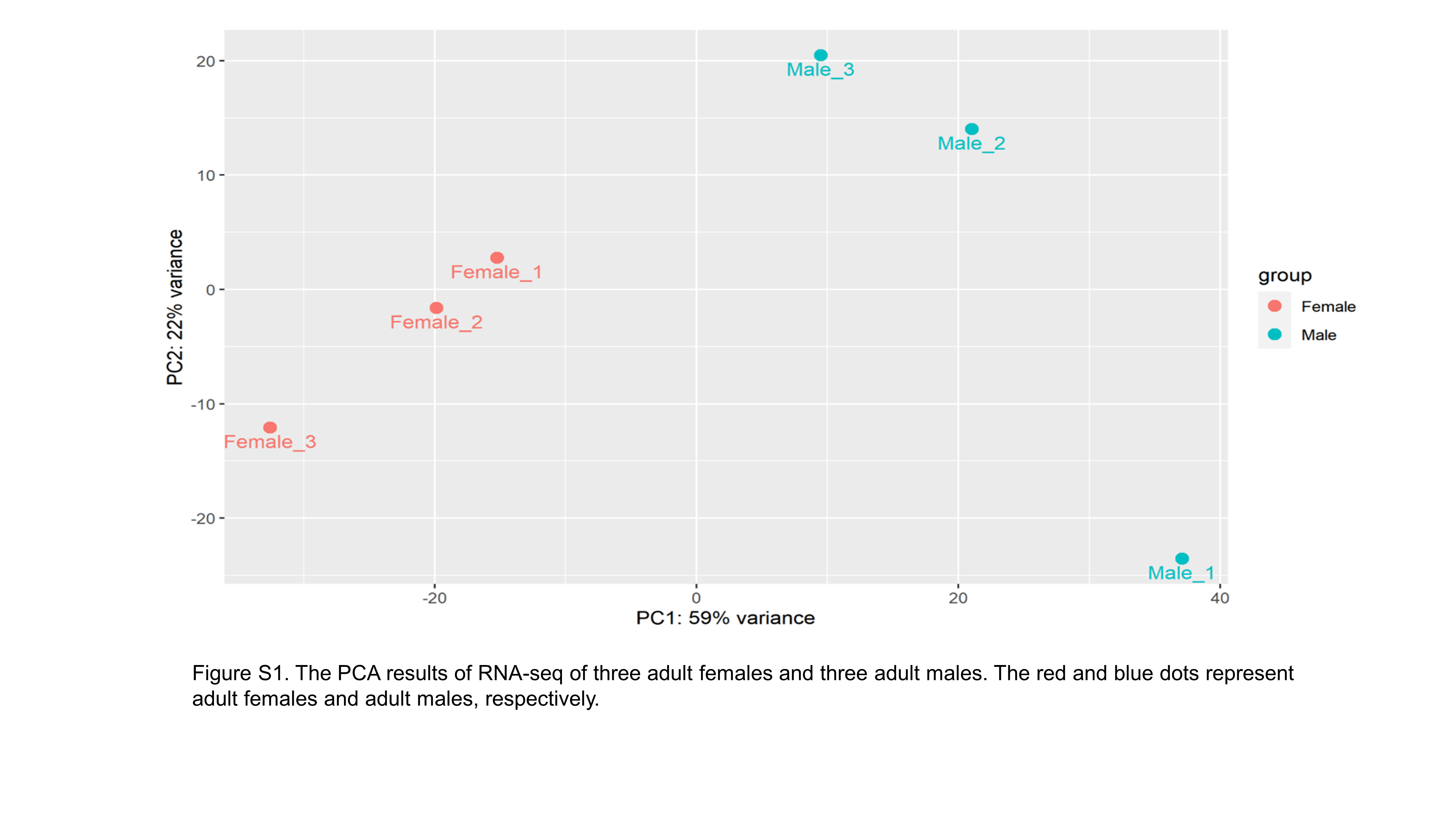

Supplement: Supplementary file 1 [file insects-14-00620-s001.zip › Figure_S1.tif]
